# Hepatitis B and C virus infections and the risk of biliary tract cancers: a meta-analysis of observational studies

**DOI:** 10.1186/s13027-022-00457-9

**Published:** 2022-08-27

**Authors:** Yizhou Wang, Ye Yuan, Dongqing Gu

**Affiliations:** 1 Department of Pathology, The Third Hospital of Mianyang, Sichuan Mental Health Center, Mianyang, 621000 China; 2 Department of Infectious Diseases, First Affiliated Hospital, Army Medical University, 30 Gaotanyan Street, Shapingba District, Chongqing, 400038 China

**Keywords:** Hepatitis B virus, Hepatitis C virus, Biliary tract cancers, Cholangiocarcinoma

## Abstract

**Background:**

Both hepatitis B virus (HBV) and hepatitis C virus (HCV) infections are important risk factors for hepatocellular carcinoma. However, their effect on other hepatobiliary cancers, such as biliary tract cancers (BTCs), is not well established. We aimed to investigate associations between HBV or HCV infection and BTCs risk by conducting a systematic review and meta-analysis.

**Methods:**

We searched PubMed to identify all relevant articles published before June 9, 2021. Meta-analysis was performed to calculate pooled odds ratios (ORs) and corresponding 95% confidence intervals (CIs). The meta-analysis was evaluated by heterogeneity testing, sensitivity analyses, and publication bias assessment.

**Results:**

In total, 48 articles involving 69,723 cases and 4,047,574 controls were obtained to calculate the associations between HBV or HCV infection and the risk of BTCs. We found that both HBV and HCV infections were associated with the risk of BTCs, with pooled ORs of 2.16 (95% CI 1.73–2.69) and 2.12 (95% CI 1.62–2.77), respectively. Subgroup analyses by ethnicity suggested that HBV infection could increase the risk of BTCs in both Asian (OR = 2.29, 95% CI 1.76–2.97) and Caucasian (OR = 1.80, 95% CI 1.18–2.75) populations. In addition, HCV infection resulted in a higher increased risk of BTCs in Caucasian populations than in Asian populations (OR = 3.93 vs*.* 1.51, *P* = 0.014). In particular, significantly increased risks of intrahepatic cholangiocarcinoma (ICC) were identified in individuals with HBV (OR = 3.96, 95% CI 3.05–5.15) or HCV infection (OR = 2.90, 95% CI 2.07–4.08).

**Conclusions:**

This study suggests that both HBV and HCV infections are risk factors for BTCs, particularly ICC, highlighting the necessity of cancer screening for BTCs in patients with either HBV or HCV infection.

**Supplementary Information:**

The online version contains supplementary material available at 10.1186/s13027-022-00457-9.

## Introduction

Viral hepatitis infections remain a major public health problem worldwide, affecting hundreds of millions of people; an estimated 257 million people are living with hepatitis B virus (HBV), and 71 million people are living with hepatitis C virus (HCV) [1]. Most deaths from viral hepatitis have been reported to be due to HBV and HCV infections.

Both HBV and HCV are potential risk factors for liver cancer, non-Hodgkin lymphoma, multiple myeloma, and thyroid cancer [2–6]. Importantly, HBV and HCV might be causal risk factors for hepatocellular carcinoma (HCC). HBV was estimated to be the cause of 53% of HCC cases, while HCV was estimated to be the cause of 25% of HCC cases worldwide [7]. However, the relationship between HBV or HCV infections and other hepatobiliary cancers, such as biliary tract cancers (BTCs), remains poorly understood.

Historically, BTCs are classified into cancers of the gallbladder, intrahepatic bile duct, extrahepatic bile duct, and ampulla of Vate, which are relatively rare but highly fatal malignancies [8, 9]. Prior studies investigating associations of HBV or HCV infection with BTC risk have yielded inconsistent or inconclusive results [10–15]. Several meta-analyses have been performed on this topic; however, these meta-analyses mainly focused on one cancer type or one hepatitis virus type [16–19]. Research systematically summarizing the relationship between them is lacking. Therefore, we aim to conduct a systematic review and meta-analysis to investigate the role of HBV or HCV infection in the BTCs risk, as well as its subtypes. Furthermore, subgroup analyses by ethnicity were performed since different endemic hepatitis virus types in different regions may determine the diversity of risk factors for BTCs.

## Materials and methods

This meta-analysis followed the guidelines of the Preferred Reporting Items for Systematic Reviews and Meta-Analyses (PRISMA) statement and Meta-analysis Of Observational Studies in Epidemiology (MOOSE) [20, 21]. The review protocol was registered in PROSPERO (CRD42021261784).

### Literature search

A comprehensive literature search of related studies was conducted using PubMed (published on or before June 9, 2021) with the keywords “hepatitis B virus or hepatitis C virus or HBV or HCV” and “cholangiocarcinoma or CCA or ECC or ICC or bile duct cancer or bile duct carcinoma or biliary tract cancer or gallbladder cancer or gallbladder neoplasm” as query terms. The titles and abstracts of the studies, or full texts if necessary, were reviewed to identify all relevant publications. Additionally, the reference lists of all included studies were manually reviewed, as were references from reviews and meta-analyses of relevant potential studies.

### Inclusion and exclusion criteria

Inclusion criteria for studies investigating the associations between HBV or HCV infections and the BTCs risk were as follows: (1) a case‒control or cohort study design; (2) exposure to HBV or HCV infection (with serum HBsAg and hepatitis C antibody used as the positive markers of hepatitis virus infection); (3) the outcome of interest was BTC, cholangiocarcinoma (CCA), intrahepatic cholangiocarcinoma (ICC), extrahepatic cholangiocarcinoma (ECC), and gallbladder cancer (GBC) (the outcome was defined according to reports in the original papers); (4) risk estimates with 95% confidence intervals (CIs) or data to calculate them were provided; and (5) publication in English. Abstracts, reviews, letters, case reports and studies that did not provide sufficient data to calculate risk estimates were excluded. Two investigators independently selected studies, and any discrepancies were resolved by consensus.

### Data extraction

The following information was extracted from each study: first author, year of publication, country, ethnicity, cancer type, study design, source population, numbers of cases and controls, odds ratios (ORs), relative risks (RRs), adjusted odds ratios (aORs) and corresponding 95% CIs, adjustments, sample sizes and follow-up years (only for cohort studies).

### Statistical analysis

Statistical analyses were performed with Stata version 15 (Stata, College Station, TX, USA), and two-sided *P* values less than 0.05 (bold values) were considered significant unless otherwise stated. Associations between hepatitis virus infection and BTC risk were evaluated by ORs and corresponding 95% CIs using a random effects model (DerSimonian Laird). The significance of the overall OR was determined by the Z-test.

The meta-analysis was evaluated by heterogeneity testing, sensitivity analyses, and publication bias assessment. Statistical heterogeneity among the studies was assessed by the Cochran Q statistic (*P* values less than 0.10 were considered indicative of statistically significant heterogeneity) and *I*^2^ statistic (*I*^2^ values less than 25% represented mild heterogeneity, values between 25 and 50% represented moderate heterogeneity, and values greater than 50% represented large heterogeneity). Subgroup analyses and meta-regression analyses were conducted by ethnicity and study design to explore potential sources of heterogeneity. In addition, sensitivity analyses were performed to investigate the influences of single studies on the overall risk estimate by removing one study at a time. Potential publication bias was evaluated using both Begg’s funnel plot and Egger’s test, and *P* values less than 0.10 were considered to be statistically significant [22, 23].

## Results

### Literature search results and study characteristics

**Fig. 1:**
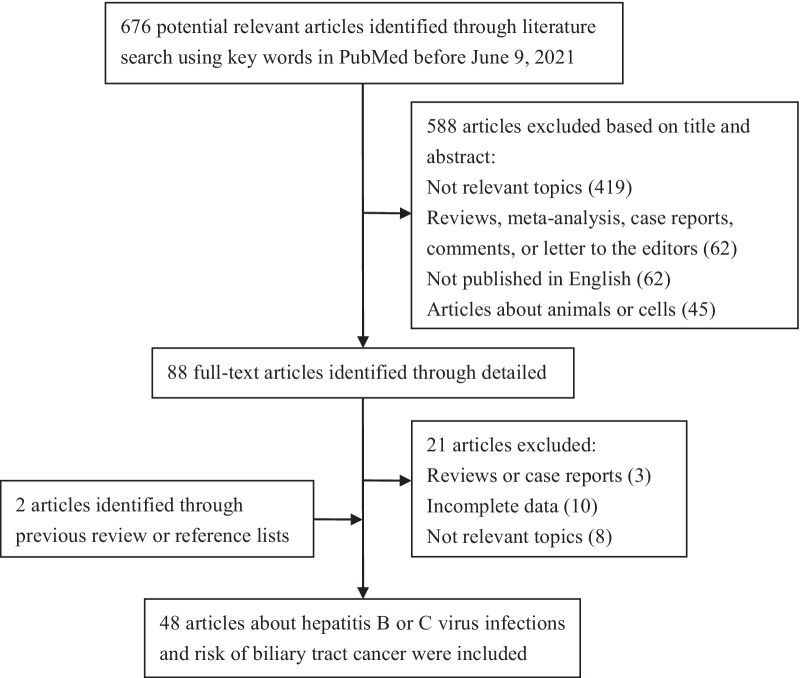
The literature search flow chart

The literature search and selection process is summarized in Fig. 1. The comprehensive search strategy generated 676 potentially relevant studies, 88 of which were considered of potential value, and their full texts were retrieved for detailed evaluation. Furthermore, 21 studies were subsequently excluded, and two additional studies were included from the references reviewed. Finally, 48 studies met our inclusion criteria and were included in our meta-analysis.

The characteristics of the included studies are summarized in Additional file 1: Table S1. Forty-eight studies investigated associations between HBV or HCV infections and the BTCs risk; of these studies, 25 studies were conducted in China, 11 in the United States, six in Korea, two in Thailand, two in Japan, one in Europe, and one in Italy. Specifically, 44 studies involving 54,521 cases and 3,733,577 controls explored the association between HBV infection and BTCs risk, and 32 studies with a total of 44,749 cases and 1,947,161 controls estimated the association between HCV infection and BTCs risk.

### HBV infection and the risk of BTC

**Fig. 2:**
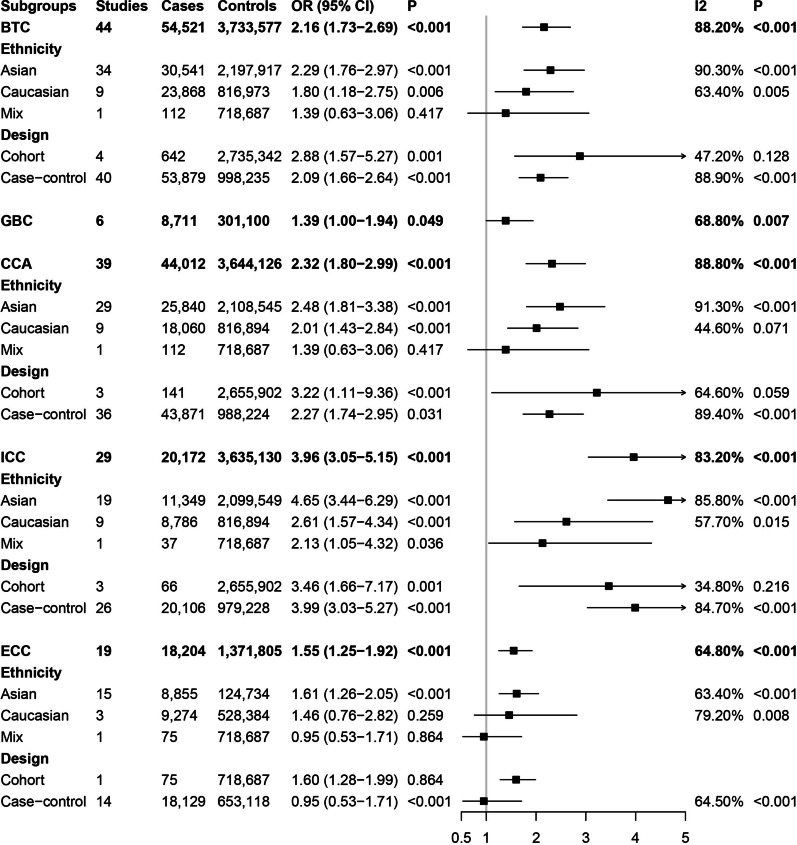
Forest plots of association between hepatitis B virus infection and biliary tract cancer risk

The HBV infection and BTCs risk results are shown in Fig. 2. We found a positive association between HBV infection and BTCs risk (OR = 2.16, 95% CI 1.73–2.69). Furthermore, we analyzed the role of HBV infection in the subtypes of BTC. The results showed that HBV infection could increase the risk of GBC (OR = 1.39, 95% CI 1.00–1.94) and CCA (OR = 2.32, 95% CI 1.80–2.99). In addition, HBV infection was associated with increased risks of ICC (OR = 3.96, 95% CI 3.05–5.15) and ECC (OR = 1.55, 95% CI 1.25–1.92).

Importantly, subgroup analyses by ethnicity suggested that HBV infection could increase the risk of BTCs in both Asian (OR = 2.29, 95% CI 1.76–2.97) and Caucasian (OR = 1.80, 95% CI 1.18–2.75) populations. Otherwise, HBV infection was associated with an increased risk of CCA in Asian populations, with a pooled OR of 2.48 (95% CI 1.81–3.38), and the pooled OR was 2.01 (95% CI 1.43–2.84) in Caucasian populations. In addition, we found that HBV infection could increase the risk of ICC (OR = 4.65, 95% CI 3.44–6.29) and ECC (OR = 1.61, 95% CI 1.26–2.05) in Asian populations. However, no association was observed between HBV infection and ECC in Caucasian populations.

### HCV infection and the risk of BTC

**Fig. 3:**
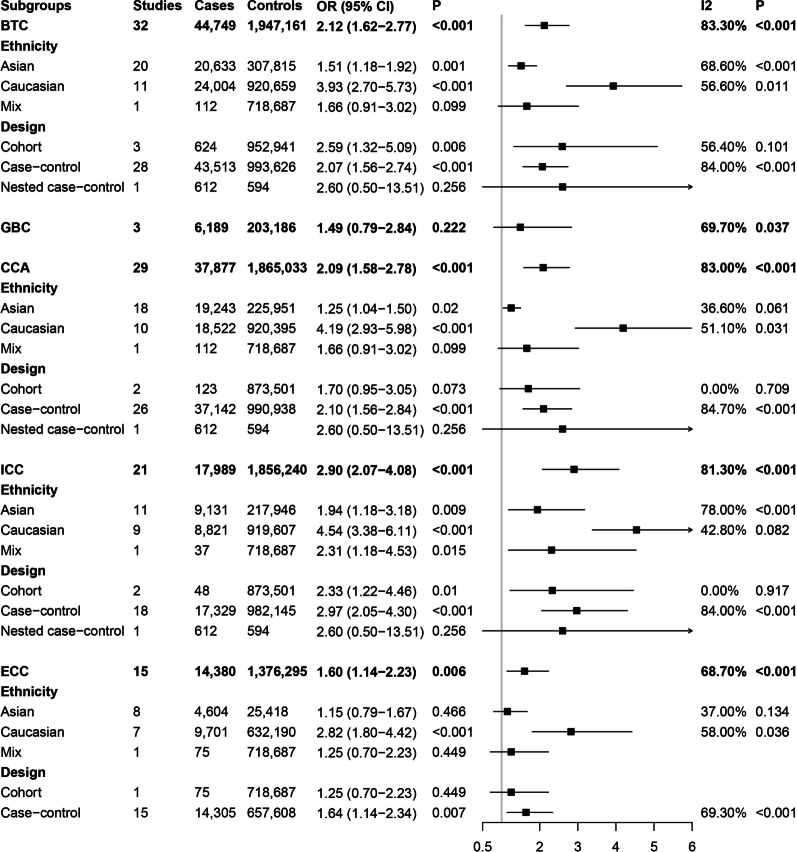
Forest plots of association between hepatitis C virus infection and biliary tract cancer risk

A significant association was found between HCV infection and BTCs risk (OR = 2.12, 95% CI 1.62–2.77) (Fig. 3). The results of our meta-analysis also showed that HCV infection could increase the risk of CCA (OR = 2.09, 95% CI 1.58–2.78), including both ICC (OR = 2.90, 95% CI 2.07–4.08) and ECC (OR = 1.60, 95% CI 1.14–2.23). However, no association was observed between HBV infection and GBC (OR = 1.49, 95% CI 0.79–2.84).

Subgroup analyses by ethnicity showed that HCV infection could increase the risk of BTCs in both Asian (OR = 1.51, 95% CI 1.18–1.92) and Caucasian (OR = 3.93, 95% CI 2.70–5.73) populations. In addition, HCV infection was associated with an increased risk of CCA (Caucasian populations: OR = 4.19, 95% CI 2.93–5.98; Asian populations: OR = 1.25, 95% CI 1.04–1.50), especially ICC (Caucasian populations: OR = 4.54, 95% CI 3.38–6.11; Asian populations: OR = 1.94, 95% CI 1.18–3.18) in these two populations. However, an increased risk of ECC with HCV infection was observed only in Caucasian populations (OR = 2.82, 95% CI 1.80–4.42) and not in Asian populations (OR = 1.15, 95% CI 0.79–1.67).

### Heterogeneity and meta-regression analyses

Large heterogeneity was found in our study, and therefore, both subgroup analyses and meta-regression analyses were conducted by ethnicity and study design to explore the potential sources of heterogeneity (Table 1). We found that only ethnicity was a significant source of heterogeneity for the association between HCV infection and BTCs risk (*P* = 0.014), CCA (*P* = 0.005), including ICC (*P* = 0.019) and ECC (*P* = 0.025). Ethnicity could explain 18% of the heterogeneity in the HCV infection–BTCs risk association, 26% of the heterogeneity in the HCV infection–CCA risk association, 46% of the heterogeneity in the HCV infection–ICC risk association, and 35% of the heterogeneity in the HCV infection–ECC risk association.

**Table 1:** Meta-regression analysis

Variables	Coefficient	Standard error	*P* value	95% CI	Proportion of explained sources of heterogeneity
*HBV and BTC*					
Ethnicity	0.325	0.241	0.186	− 0.812 to 0.162	–
Study design	0.484	0.402	0.236	− 0.328 to 1.296	–
*HBV and CCA*					
Ethnicity	− 0.350	.0263	0.191	− 0.883 to 0.182	–
Study design	0.551	0.520	0.296	− 0.502 to 1.605	–
*HBV and ICC*					
Ethnicity	0.363	0.380	0.348	− 0.419 to 1.145	–
Study design	− 0.307	0.642	0.636	− 1.627 to 1.013	–
*HBV and ECC*					
Ethnicity	− 0.032	0.292	0.914	− 0.650 to 0.587	–
Study design	0.460	0.710	0.526	− 1.966 to 1.045	–
*HCV and BTC*					
Ethnicity	0.578	0.222	**0.014**	0.125 to 1.032	18%
Study design	0.073	0.352	0.837	− 0.793 to 0.647	–
*HCV and CCA*					
Ethnicity	0.675	0.222	**0.005**	0.219 to 1.130	26%
Study design	0.006	0.420	0.989	− 0.855 to 0.867	–
*HCV and ICC*					
Ethnicity	0.595	0.232	**0.019**	− 1.081 to − 0.109	46%
Study design	− 0.132	0.399	0.745	− 0.968 to 0.704	–
*HCV and ECC*					
Ethnicity	− 0.594	0.234	**0.025**	− 1.100 to − 0.089	35%
Study design	0.249	0.667	0.715	− 1.689 to 1.191	–

CCA, cholangiocarcinoma; CI, confidence interval; ECC, extrahepatic cholangiocarcinoma; HBV, hepatitis B virus; HCV, hepatitis C virus; ICC, intrahepatic cholangiocarcinoma

### Sensitivity analysis and publication bias

Sensitivity analysis was performed to evaluate the stability of the results, and we found that removing a single study could not significantly change the pooled ORs and 95% CIs (data not shown), indicating that any single study had little impact on the overall ORs. In addition, the funnel plot appeared generally symmetrical (Additional file 1: Figs. S1, S2), and no significant publication bias was detected using either Begg’s test or Egger’s test in our study (*P* > 0.1 in all the analyses, except for the associations between HBV and BTC risk and HBV and CCA risk).

## Discussion

Our study included 44 case‒control studies and four cohort studies exploring the associations between HBV infection and BTCs risk with a total of 54,521 cases and 3,733,577 controls and between HCV infection and BTCs risk in 44,749 cases and 1,947,161 controls. To the best of our knowledge, this study is the largest and most comprehensive meta-analysis estimating the relationship between HBV or HCV infection and BTCs to date. The results showed that both HBV and HCV infections are risk factors for BTCs, highlighting the necessity of cancer screening for BTCs in individuals with HBV or HCV infection.

The present study found that compared with individuals with non-HBV infections, patients with HBV infection were associated with a 2.16-fold increased risk of BTCs. In particular, HBV infection was associated with approximately 1.39- to 3.96-fold higher risks of BTCs (GBC: 1.39, ICC: 3.96, and ECC: 1.55, respectively). In addition, patients with HCV infection had an approximately 2.12-fold increased risk of BTCs, with a 2.90-fold increased risk of ICC and a 1.60-fold increased risk of ECC. However, no evidence of an association between HCV infection and GBC was found. These results suggest the importance of considering ICC and ECC separately since both HBV and HCV infections have a higher risk of ICC than ECC. In addition, these findings highlight aetiologic heterogeneity across BTCs, and the disparity might be explained by the different anatomic locations. Both HBV and HCV prefer to replicate in hepatocytes, which could lead to liver cirrhosis and HCC [24]. Since hepatocytes and cholangiocytes differentiate from a common hepatic progenitor cell, both HBV and HCV can be postulated to induce carcinogenesis of CCA through a similar mechanism as in HCC [25, 26].

Subgroup analyses by ethnicity showed that both Causation and Asian populations had comparably higher increased risks of BTCs with HBV infection (OR: 2.29 vs. 1.80, *P* = 0.186), whereas HCV infection was associated with a higher increased risk of BTCs in Causation populations than in Asian populations (OR: 3.93 vs. 1.51, *P* = 0.014). HBV infection, but not HCV infection, was reported to be associated with BTC in some Asian countries, such as China, where HBV infection is highly endemic [26–28]. However, HCV-related ICC was more likely to occur in regions where HCV was highly endemic, such as Western countries, the United States and Japan [29–31]. These results indicated that different endemic hepatitis virus types in different regions may determine the diversity of risk factors for BTCs.

The pathogenesis of HBV and HCV infections in BTC development has not yet been clarified. HBV x protein, which is encoded by the HBV x gene, is well known as the major causative factor for hepatocarcinogenesis [32–35]. Long-term high expression of several viral oncoproteins, mostly the HBV x protein and HCV core protein, may be involved in the tumorigenic process [32, 36]. On the other hand, HCV C protein, which is encoded by the HCV core gene, was reported to have a critical role in the invasion and metastasis of CCA by inducing epithelial-mesenchymal transition (EMT) in a CCA cell line [37]. In addition, persistent chronic inflammation of biliary epithelia caused by chronic HBV or HCV infection is probably involved in cholangiocarcinogenesis [38, 39].

However, several limitations in this meta-analysis should be considered. First, we did not search for carcinoma of Vaters Ampulla (AVC) in the literature since cancers arising from the ampulla of Vater are sometimes included under the term BTCs. Histologically, such cancers can be pancreatobiliary, intestinal, or mixed tumours and account for only 0.2% of gastrointestinal cancers [40]. In addition, fewer studies reported this cancer alone. Second, the included studies were all observational studies, which might introduce selection and recall biases. Third, in different studies, the definitions of the outcomes were usually different (such as histological diagnosis, ICD-9, or ICD-10), which might have implications for pooled estimates. Fourth, significant heterogeneity was observed in our meta-analyses, which lowers the reliability of the overall ORs, although subgroup analyses and meta-regression analyses were conducted to explore potential sources of heterogeneity; however, only ethnicity could explain part of the heterogeneity. Fifth, although we extracted the most fully adjusted risk estimates, adjusted confounders varied among the included studies, and some potential confounding factors (such as age, sex, race, smoking status, and alcohol consumption) may influence the relationship between hepatitis virus infection and BTCs risk. Finally, potential publication bias was observed in our study, often because studies with positive results were more likely to be published than studies with negative results.

In conclusion, this meta-analysis shows that both HBV and HCV infections are risk factors for BTCs, especially ICC. Furthermore, we found that both Asian and Causation populations have comparably higher risks of HBV-related BTCs, whereas Causation populations have a higher risk of HCV-related BTCs. Findings from our study suggest an important role of hepatitis virus infection in the pathogenesis of BTCs, highlighting the necessity of cancer screening for BTCs in individuals with HBV or HCV infection.

## Supplementary Information

**Additional file 1: Table S1**. Characteristics of studies included in the meta-analysis. **Figure S1**. Funnel plot of studies evaluating the association between hepatitis B virus (HBV) and biliary tract cancer risk. (a) HBV and biliary tract cancer, (b) HBV and cholangiocarcinoma, (c) HBV and intrahepatic cholangiocarcinoma, (d) HBV and extrahepatic cholangiocarcinoma risk. **Figure S2**. Funnel plot of studies evaluating the association between hepatitis C virus (HCV) and biliary tract cancer risk. (a) HCV and biliary tract cancer, (b) HCV and cholangiocarcinoma, (c) HCV and intrahepatic cholangiocarcinoma, (d) HCV and extrahepatic cholangiocarcinoma risk.

## Data Availability

The data that support the findings of this study are available from the corresponding author upon reasonable request.

## Abbreviations

AVC: Carcinoma of Vaters Ampulla
BTCs: Biliary Tract Cancers
CCA: Cholangiocarcinoma
CI: Confidence interval
ECC: Extrahepatic cholangiocarcinoma
HBV: Hepatitis B virus
HCC: Hepatocellular carcinoma
HCV: Hepatitis C virus
ICC: Intrahepatic cholangiocarcinoma
GBC: Gallbladder cancer
OR: Odds ratio
