# Supplementary material for: Hepatitis B and C virus infections and the risk of biliary tract cancers: a meta-analysis of observational studies
Source: Infect Agent Cancer. 2022 Aug 27;17:45. doi: 10.1186/s13027-022-00457-9 (PMC9420284; doi:10.1186/s13027-022-00457-9)
Supplement: Supplementary file 1 — Additional file 1: Table S1. Characteristics of studies included in the meta-analysis. Figure S1. Funnel plot of studies evaluating the association between hepatitis B virus (HBV) and biliary tract cancer risk. (a) HBV and biliary tract cancer, (b) HBV and cholangiocarcinoma, (c) HBV and intrahepatic cholangiocarcinoma, (d) HBV and extrahepatic cholangiocarcinoma risk. Figure S2. Funnel plot of studies evaluating the association between hepatitis C virus (HCV) and biliary tract cancer risk. (a) HCV and biliary tract cancer, (b) HCV and cholangiocarcinoma, (c) HCV and intrahepatic cholangiocarcinoma, (d) HCV and extrahepatic cholangiocarcinoma risk. [file 13027_2022_457_MOESM1_ESM.docx]

**Additional file 1:**

**Hepatitis B and C Virus Infections associated with Risk of Biliary Tract Cancer: a Meta-Analysis of Observational Studies**

Yizhou Wang^1^, Ye Yuan^2^, and Dongqing Gu^2*^

**Corresponding author contact information:**

**Dongqing Gu, M.D., Ph.D.**

Department of Epidemiology and Biostatistics

First Affiliated Hospital

Army Medical University

30 Gaotanyan Street, Shapingba District, Chongqing 400038, China

Phone: 86-23-6875-4311, Fax: 86-23-6875-4311

E-mail: [dongqing.gu@vip.163.com](file:///D:\project\HBV_HCV%20AND%20CC\20210609\dongqing.gu@vip.163.com)

**Affiliations:**

^1^Department of Pathology, The Third Hospital of Mianyang，Sichuan Mental Health Center,Mianyang 621000, China

^2^Department of Epidemiology and Biostatistics, First Affiliated Hospital, Army Medical University, Chongqing 400038, China

**Additional file 1: Table S1. Characteristics of studies included in the meta-analysis.**

| **Study** | **Country** | **Ethnicity** | **Design** | **Cancer type** | **Cases (n)** | **Controls (n)** | **Hepatitis virus** | **OR (95% CI)** |
| --- | --- | --- | --- | --- | --- | --- | --- | --- |
| Parkin(1991)(1) | Thailand | Asian | Case-control | CCA | 103 | 103 | HBV | 1.00 (0.50-1.80) |
| Shin(1996)(2) | Korea | Asian | Case-control | CCA | 41 | 406 | HBV | 1.30 (0.30-5.30) |
|  |  |  |  |  |  |  | HCV | 3.90 (0.90-17.10) |
| Donato(2001)(3) | Italy | Caucasian | Case-control | ICC | 26 | 824 | HBV | 2.70 (0.40-18.50) |
|  |  |  |  |  |  |  | HCV | 9.70 (1.60-58.90) |
| Yamamoto(2004)(4) | Japan | Asian | Case-control | ICC | 50 | 205 | HBV | 1.84 (0.34-10.11) |
|  |  |  |  |  |  |  | HCV | 6.02 (1.51-24.10) |
| Shaib(2005)(5) | US | Caucasian | Case-control | ICC | 625 | 90,834 | HBV | 0.80 (0.10-5.90) |
|  |  |  |  |  |  |  | HCV | 5.20 (2.10-12.80) |
| Choi(2006)(6) | Korea | Asian | Case-control | ICC | 51 | 51 | HBV | 0.80 (0.20-3.02) |
|  |  |  |  |  |  |  | HCV | 1.00 (0.04-25.26) |
| Shaib(2007)(7) | US | Caucasian | Case-control | ICC | 83 | 236 | HBV | 2.90 (0.10-236.80) |
|  |  |  |  |  |  |  | HCV | 7.90 (1.30-84.50) |
|  |  |  |  | ECC | 163 | 236 | HCV | 2.80 (0.30-35.10) |
| Welzel(2007)(8) | US | Caucasian | Case-control | ICC | 535 | 102,782 | HCV | 4.40 (1.40-14.00) |
|  |  |  |  | ECC | 549 | 102,782 | HCV | 1.50 (0.20-11.00) |
| Hsing(2008)(9) | China | Asian | Case-control | GBC | 234 | 762 | HBV | 1.30 (0.70-2.40) |
|  |  |  |  | ECC | 134 | 762 | HBV | 2.40 (1.20-4.50) |
|  |  |  |  | AVC | 49 | 762 | HBV | 1.20 (0.30-4.30) |
|  |  |  |  | GBC | 234 | 762 | HCV | 0.60 (0.20-2.20) |
|  |  |  |  | ECC | 134 | 762 | HCV | 0.80 (0.20-3.40) |
|  |  |  |  | AVC | 49 | 762 | HCV | 1.00 (0.10-7.50) |
| Zhou(2008)(10) | China | Asian | Case-control | ICC | 312 | 428 | HBV | 8.88 (5.97-13.19) |
|  |  |  |  |  |  |  | HCV | 0.93 (0.28-3.10) |
| Lee(2008)(11) | Korean | Asian | Case-control | ICC | 622 | 2,488 | HBV | 2.30 (1.60-3.30) |
|  |  |  |  |  |  |  | HCV | 1.00 (0.50-1.90) |
| El-Serag(0009)(12) | US | Mix | Cohort | ICC | 37 | 718,687 | HBV | 2.13 (1.05-4.31) |
|  |  |  |  |  |  |  | HCV | 2.31 (1.18-4.54) |
|  |  |  |  | ECC | 75 | 718,687 | HBV | 0.95 (0.53-1.71) |
|  |  |  |  |  |  |  | HCV | 1.25 (0.70-2.22) |
| Lee(2009)(13) | China | Asian | Case-control | ICC | 106 | 106 | HBV | 4.99 (2.78-8.95) |
|  |  |  |  |  |  |  | HCV | 2.71 (1.16-6.32) |
| Tao(2009)(14) | China | Asian | Case-control | ICC | 61 | 380 | HBV | 18.10 (7.50-44.00) |
|  |  |  |  | ECC | 129 | 380 | HBV | 1.70 (0.80-3.60) |
| Tanaka(2010)(15) | Japan | Asian | Cohort | ICC | 11 | 154,814 | HBV | 8.56 (1.33-55.20) |
|  |  |  |  |  |  |  | HCV | 2.63 (0.25-27.73) |
| Zhou(2010)(16) | China | Asian | Case-control | ICC | 317 | 634 | HBV | 9.67 (6.33-14.77) |
| Srivatanakul(2010)(17) | Thailand | Asian | Case-control | CCA | 106 | 106 | HBV | 4.69 (0.98-22.47) |
| Peng(2011)(18) | China | Asian | Case-control | ICC | 98 | 196 | HBV | 2.75 (1.27-5.95) |
| Cai(2011)(19) | China | Asian | Case-control | ECC | 313 | 608 | HBV | 1.19 (0.70-2.03) |
|  |  |  |  |  |  |  | HCV | 0.44 (0.13-1.53) |
| Fwu(2011)(20) | China | Asian | Cohort | ICC | 18 | 1,782,401 | HBV | 4.80 (1.88-12.20) |
| Welzel(2011)(21) | US | Caucasian | Case-control | ICC | 743 | 195,953 | HBV | 3.07 (1.43-6.58) |
|  |  |  |  |  |  |  | HCV | 8.05 (5.08-12.75) |
| Wu(2012)(22) | China | Asian | Case-control | CCA | 66 | 52 | HBV | 9.38 (2.06-42.59) |
|  |  |  |  | ICC | 23 | 52 | HBV | 38.89 (7.52-201.05) |
|  |  |  |  | ECC | 43 | 52 | HBV | 4.05 (0.77-21.23) |
| Liu(2011)(23) | China | Asian | Case-control | ICC | 87 | 228 | HBV | 1.05 (0.39-2.81) |
|  |  |  |  |  |  |  | HCV | 0.87 (0.09-18.50) |
| Gong(2012)(24) | China | Asian | Case-control | BTC | 826 | 751 | HBV | 0.94 (0.56-1.58) |
| Qu(2012)(25) | China | Asian | Case-control | ECC | 305 | 480 | HBV | 2.83 (1.33-6.04) |
|  |  |  |  | ECC | 139 | 214 | HCV | 0.76 (0.28-2.07) |
| Wu(2012)(26) | China | Asian | Case-control | ICC | 102 | 835 | HBV | 10.52 (5.94-18.62) |
|  |  |  |  | ECC | 86 | 835 | HBV | 2.08 (0.84-5.17) |
|  |  |  |  | GBC | 93 | 809 | HBV | 2.36 (1.00-5.57) |
| Chaiteerakij(2013)(27) | US | Caucasian | Nested case-control | ICC | 612 | 594 | HBV | 0.97 (0.20-4.83) |
|  |  |  |  |  |  |  | HCV | 2.60 (0.50-13.50) |
| Fedirko(2013)(28) | Europeans | Caucasian | Case-control | ICC | 35 | 69 | HBV | 0.65 (0.06-6.46) |
|  |  |  |  | BTC | 133 | 264 | HBV | 0.48 (0.13-1.75) |
|  |  |  |  | BTC | 133 | 264 | HCV | 2.01 (0.40-10.09) |
| Zhou(2013)(29) | China | Asian | Case-control | ECC | 239 | 478 | HBV | 1.50 (0.89-2.52) |
| Chang(2013)(30) | China | Asian | Case-control | ICC | 2,978 | 11,912 | HBV | 3.50 (2.90-4.30) |
|  |  |  |  |  |  |  | HCV | 3.50 (2.70-4.40) |
|  |  |  |  | ECC | 2,179 | 8,716 | HBV | 2.60 (2.00-3.40) |
|  |  |  |  |  |  |  | HCV | 1.80 (1.30-2.50) |
| Lee(2015)(31) | Korea | Asian | Case-control | ECC | 81 | 162 | HBV | 1.92 (0.71-8.07) |
|  |  |  |  |  |  |  | HCV | 2.50 (0.67-9.31) |
| Lee(2015)(32) | Korea | Asian | Case-control | CCA | 276 | 552 | HBV | 4.12 (2.01-8.44) |
|  |  |  |  |  |  |  | HCV | 1.69 (0.76-3.78) |
|  |  |  |  | ICC | 193 | 386 | HBV | 2.10 (0.80-5.49) |
|  |  |  |  |  |  |  | HCV | 1.20 (0.44-3.30) |
|  |  |  |  | ECC | 83 | 166 | HBV | 5.27 (1.93-14.38) |
|  |  |  |  |  |  |  | HCV | 1.71 (0.25-11.45) |
| Peng(2015)(33) | China | Asian | Case-control | CCA | 3,174 | 3,174 | HBV | 0.93 (0.82-1.06) |
|  |  |  |  |  |  |  | HCV | 1.01 (0.86-1.19) |
| Choi(2016)(34) | US | Caucasian | Case-control | CCA | 2,395 | 4,769 | HBV | 2.78 (1.04-7.43) |
|  |  |  |  |  |  |  | HCV | 1.94 (0.95-3.95) |
|  |  |  |  | ICC | 1,169 | 4,769 | HBV | 12.90 (2.69-61.61) |
|  |  |  |  |  |  |  | HCV | 1.95 (0.75-5.11) |
|  |  |  |  | ECC | 995 | 4,769 | HBV | 0.17 (0.02-1.26) |
|  |  |  |  |  |  |  | HCV | 3.51 (1.02-12.08) |
|  |  |  |  | ECC | 231 | 4,769 | HBV | 1.32 (0.01-196.9) |
|  |  |  |  |  |  |  | HCV | 0.17 (0.01-5.17) |
| Meng(2017)(35) | China | Asian | Case-control | CCA | 55 | 926 | HBV | 1.66 (0.80-3.42) |
| Kamiza(2016)(36) | China | Asian | Cohort | BTC | 501 | 79,440 | HBV | 2.80 (1.64-4.76) |
|  |  |  |  |  |  |  | HCV | 3.81 (2.38-6.10) |
| Huang(2017)(37) | China | Asian | Case-control | ICC | 4,695 | 46,942 | HBV | 2.23 (1.80-2.76) |
|  |  |  |  |  |  |  | HCV | 1.26 (1.00-1.58) |
|  |  |  |  | ECC | 1,398 | 13,964 | HBV | 0.95 (0.58-1.56) |
|  |  |  |  |  |  |  | HCV | 0.95 (0.59-1.54) |
| Mahale(2017)(38) | US | Caucasian | Case-control | ICC | 2,936 | 200,000 | HCV | 3.40 (2.52-4.58) |
|  |  |  |  | ECC | 4,370 | 200,000 | HCV | 1.90 (1.41-2.57) |
|  |  |  |  | GBC | 5,349 | 200,000 | HCV | 1.35 (0.95-1.91) |
| Wei(2017)(39) | China | Asian | Case-control | BTC | 220 | 5,715 | HBV | 2.69 (1.93-3.74) |
| Petrick(2017)(40) | US | Caucasian | Case-control | ICC | 2,092 | 323,615 | HBV | 2.97 (1.97-4.46) |
|  |  |  |  |  |  |  | HCV | 4.67 (3.57-6.11) |
|  |  |  |  | ECC | 2,981 | 323,615 | HBV | 2.38 (1.65-3.44) |
|  |  |  |  |  |  |  | HCV | 3.18 (2.43-4.16) |
| An(2018)(41) | Korea | Asian | Case-control | CCA | 4,206 | 85,744 | HBV | M: 2.59 (1.98-3.39)  F:1.71 (1.16-2.51) |
|  |  |  |  | ICC | 1,402 | 85,744 | HBV | M:6.20 (4.69-8.19)  F:4.73 (3.02-7.42) |
|  |  |  |  | ECC | 2,786 | 85,744 | HBV | M: 0.77 (0.53-1.11)  F:1.11 (0.66-1.86) |
|  |  |  |  | GBC | 1,236 | 85,744 | HBV | M:0.94 (0.48-1.86)  F:1.06 (0.55-2.05) |
| Mahale(2018)(42) | US | Caucasian | Case-control | ICC | 3,401 | 200,000 | HBV | 1.67 (1.18-2.37) |
|  |  |  |  | GBC | 6,083 | 200,000 | HBV | 1.11 (0.80-1.54) |
|  |  |  |  | ECC | 5,067 | 200,000 | HBV | 1.34 (0.98-1.82) |
| Peng(2018)(43) | China | Asian | Case-control | CCA | 2,293 | 2,293 | HBV | 1.00 (0.85-1.17) |
|  |  |  |  |  |  |  | HCV | 1.08 (0.90-1.29) |
| Xiong(2018)(44) | China | Asian | Case-control | CCA | 303 | 606 | HBV | 2.63 (1.54-4.51) |
|  |  |  |  |  |  |  | HCV | 1.25 (0.41-3.87) |
|  |  |  |  | ICC | 136 | 606 | HBV | 5.27 (2.95-9.42) |
|  |  |  |  |  |  |  | HCV | 2.85 (0.92-8.86) |
|  |  |  |  | ECC | 167 | 606 | HBV | 0.69 (0.26-1.82) |
|  |  |  |  |  |  |  | HCV | 0.91 (0.19-4.31) |
| Zhou(2018)(45) | China | Asian | Case-control | ECC | 200 | 200 | HBV | 1.61 (0.92-2.83) |
| Lavu(2020)(46) | US | Caucasian | Case-control | ECC | 412 | 788 | HCV | 7.33 (2.94-18.31) |
| Xiong(2020)(47) | China | Asian | Case-control | GBC | 606 | 2,424 | HBV | 2.31 (1.72-3.25) |
|  |  |  |  |  |  |  | HCV | 2.65 (1.44-4.36) |
| Tian(2020)(48) | China | Asian | Case-control | ICC | 179 | 11,361 | HBV | 3.83 (2.58-5.67) |
|  |  |  |  | ECC | 602 | 11,361 | HBV | 1.72 (1.28-2.31) |
|  |  |  |  | GBC | 459 | 11,361 | HBV | 1.11 (0.73-1.68) |
|  |  |  |  | Other BTC | 49 | 11,361 | HBV | 1.15 (0.36-3.72) |

AVC: Ampulla of vater cancer; BTC: Biliary tract cancer; CCA,cholangiocarcinoma; CI, confidence interval; ECC, extrahepatic cholangiocarcinoma;

GBC: gallbladder cancer; HBV, hepatitis B virus; HCV, hepatitis C virus; ICC, intrahepatic cholangiocarcinoma; OR, odds ratio; US, United States.

**Additional file 1: Figure S1. Funnel plot of studies evaluating the association between hepatitis B virus (HBV) and biliary tract cancer risk. a. HBV and biliary tract cancer, b. HBV and cholangiocarcinoma, c. HBV and intrahepatic cholangiocarcinoma, d. HBV and extrahepatic cholangiocarcinoma risk.**

**a.**


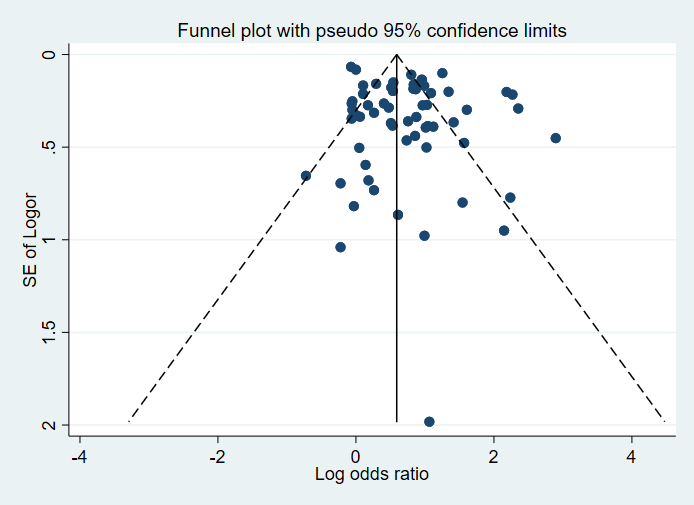


**b.**


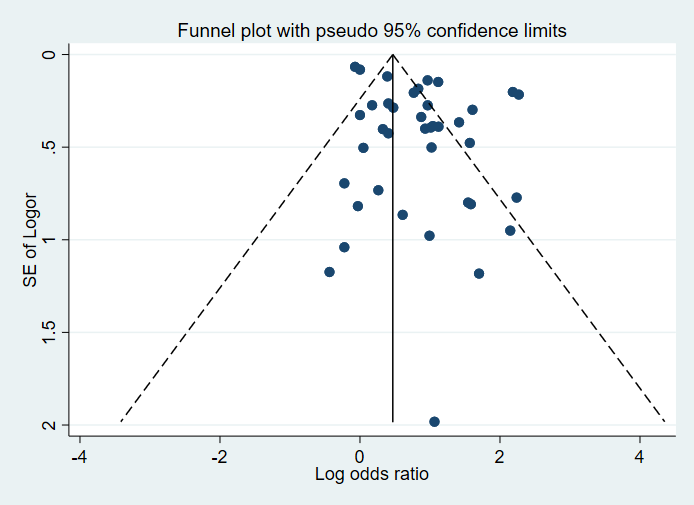


**c.**

**
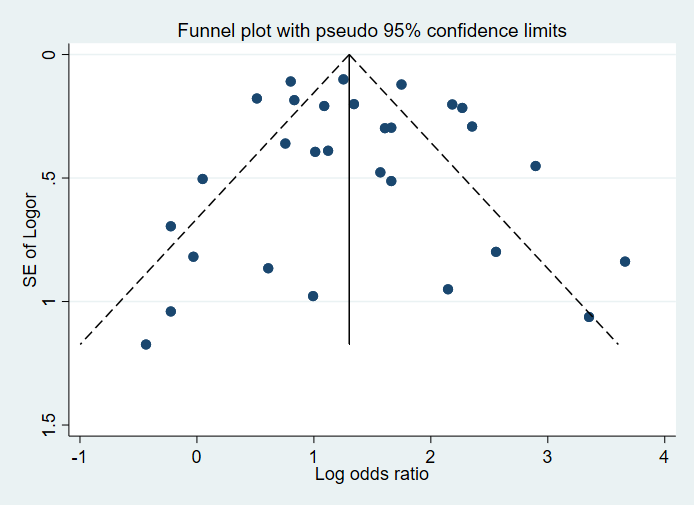
**

**d.**


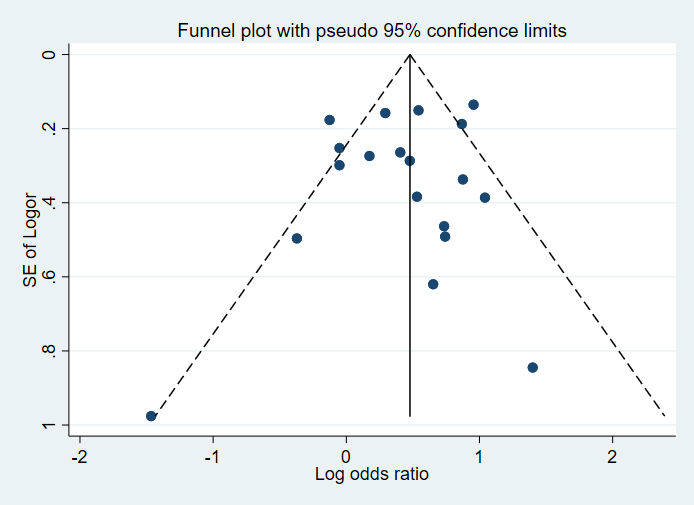


**Additional file 1: Figure S2. Funnel plot of studies evaluating the association between hepatitis C virus (HCV) and biliary tract cancer risk. a. HCV and biliary tract cancer, b. HCV and cholangiocarcinoma, c. HCV and intrahepatic cholangiocarcinoma, d. HCV and extrahepatic cholangiocarcinoma risk.**

**a.**


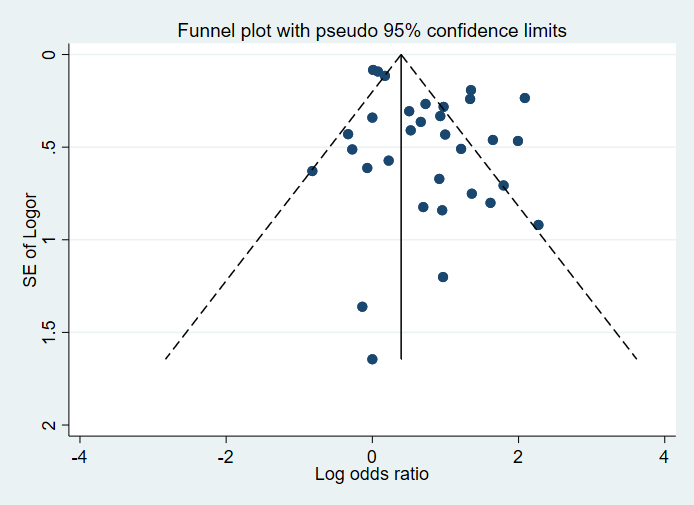


**b.**


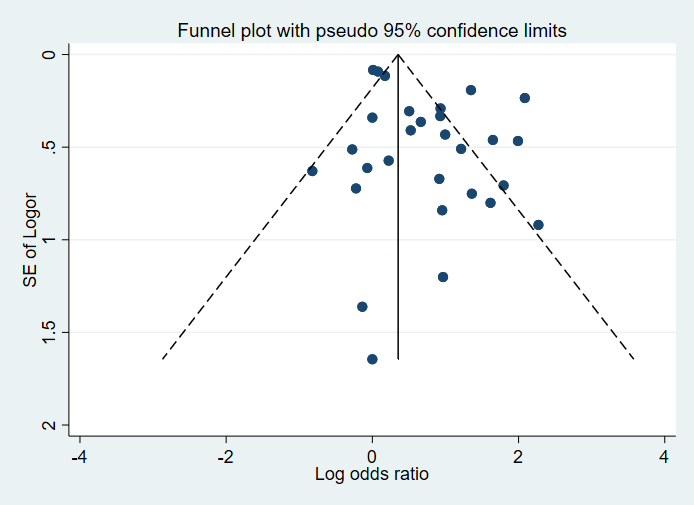


c.


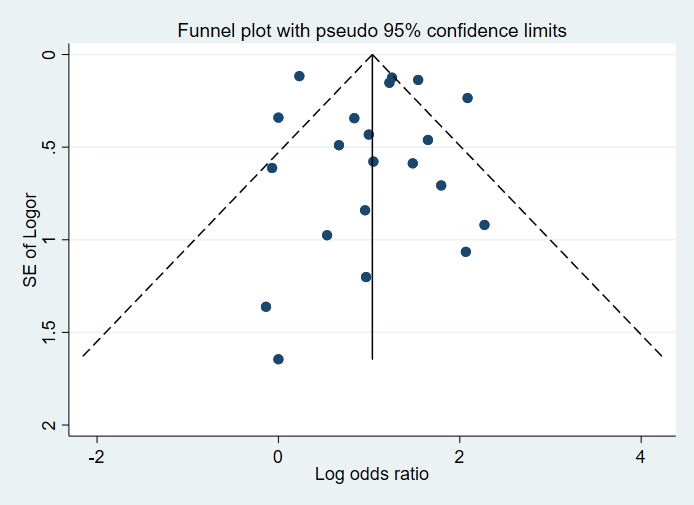


d.


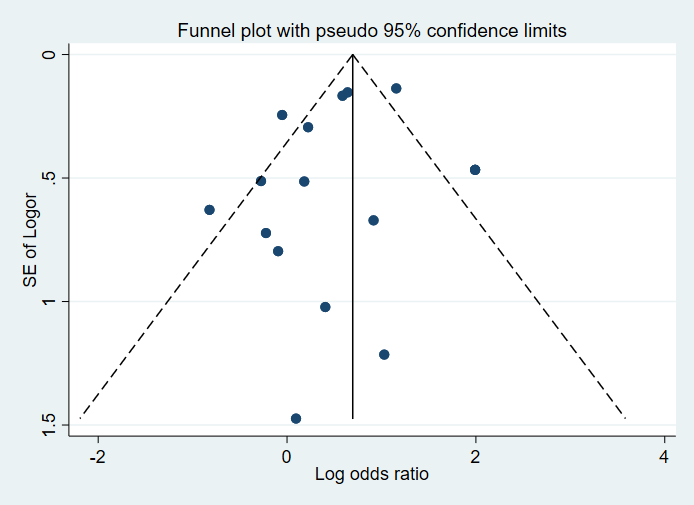


**Additional file: References**

1. Parkin DM, Srivatanakul P, Khlat M, Chenvidhya D, Chotiwan P, Insiripong S, L'Abbe KA, et al. Liver cancer in Thailand. I. A case-control study of cholangiocarcinoma. Int J Cancer 1991;48:323-328.

2. Shin HR, Lee CU, Park HJ, Seol SY, Chung JM, Choi HC, Ahn YO, et al. Hepatitis B and C virus, Clonorchis sinensis for the risk of liver cancer: a case-control study in Pusan, Korea. Int J Epidemiol 1996;25:933-940.

3. Donato F, Gelatti U, Tagger A, Favret M, Ribero ML, Callea F, Martelli C, et al. Intrahepatic cholangiocarcinoma and hepatitis C and B virus infection, alcohol intake, and hepatolithiasis: a case-control study in Italy. Cancer Causes Control 2001;12:959-964.

4. Yamamoto S, Kubo S, Hai S, Uenishi T, Yamamoto T, Shuto T, Takemura S, et al. Hepatitis C virus infection as a likely etiology of intrahepatic cholangiocarcinoma. Cancer Sci 2004;95:592-595.

5. Shaib YH, El-Serag HB, Davila JA, Morgan R, McGlynn KA. Risk factors of intrahepatic cholangiocarcinoma in the United States: a case-control study. Gastroenterology 2005;128:620-626.

6. Choi D, Lim JH, Lee KT, Lee JK, Choi SH, Heo JS, Jang KT, et al. Cholangiocarcinoma and Clonorchis sinensis infection: a case-control study in Korea. J Hepatol 2006;44:1066-1073.

7. Shaib YH, El-Serag HB, Nooka AK, Thomas M, Brown TD, Patt YZ, Hassan MM. Risk factors for intrahepatic and extrahepatic cholangiocarcinoma: a hospital-based case-control study. Am J Gastroenterol 2007;102:1016-1021.

8. Welzel TM, Graubard BI, El-Serag HB, Shaib YH, Hsing AW, Davila JA, McGlynn KA. Risk factors for intrahepatic and extrahepatic cholangiocarcinoma in the United States: a population-based case-control study. Clin Gastroenterol Hepatol 2007;5:1221-1228.

9. Hsing AW, Zhang M, Rashid A, McGlynn KA, Wang BS, Niwa S, Ortiz-Conde BA, et al. Hepatitis B and C virus infection and the risk of biliary tract cancer: a population-based study in China. Int J Cancer 2008;122:1849-1853.

10. Zhou YM, Yin ZF, Yang JM, Li B, Shao WY, Xu F, Wang YL, et al. Risk factors for intrahepatic cholangiocarcinoma: a case-control study in China. World J Gastroenterol 2008;14:632-635.

11. Lee TY, Lee SS, Jung SW, Jeon SH, Yun SC, Oh HC, Kwon S, et al. Hepatitis B virus infection and intrahepatic cholangiocarcinoma in Korea: a case-control study. Am J Gastroenterol 2008;103:1716-1720.

12. El-Serag HB, Engels EA, Landgren O, Chiao E, Henderson L, Amaratunge HC, Giordano TP. Risk of hepatobiliary and pancreatic cancers after hepatitis C virus infection: A population-based study of U.S. veterans. Hepatology 2009;49:116-123.

13. Lee CH, Chang CJ, Lin YJ, Yeh CN, Chen MF, Hsieh SY. Viral hepatitis-associated intrahepatic cholangiocarcinoma shares common disease processes with hepatocellular carcinoma. Br J Cancer 2009;100:1765-1770.

14. Tao LY, He XD, Qu Q, Cai L, Liu W, Zhou L, Zhang SM. Risk factors for intrahepatic and extrahepatic cholangiocarcinoma: a case-control study in China. Liver Int 2010;30:215-221.

15. Tanaka M, Tanaka H, Tsukuma H, Ioka A, Oshima A, Nakahara T. Risk factors for intrahepatic cholangiocarcinoma: a possible role of hepatitis B virus. J Viral Hepat 2010;17:742-748.

16. Zhou H, Wang H, Zhou D, Wang H, Wang Q, Zou S, Tu Q, et al. Hepatitis B virus-associated intrahepatic cholangiocarcinoma and hepatocellular carcinoma may hold common disease process for carcinogenesis. Eur J Cancer 2010;46:1056-1061.

17. Srivatanakul P, Honjo S, Kittiwatanachot P, Jedpiyawongse A, Khuhaprema T, Miwa M. Hepatitis viruses and risk of cholangiocarcinoma in northeast Thailand. Asian Pac J Cancer Prev 2010;11:985-988.

18. Peng NF, Li LQ, Qin X, Guo Y, Peng T, Xiao KY, Chen XG, et al. Evaluation of risk factors and clinicopathologic features for intrahepatic cholangiocarcinoma in Southern China: a possible role of hepatitis B virus. Ann Surg Oncol 2011;18:1258-1266.

19. Cai WK, Sima H, Chen BD, Yang GS. Risk factors for hilar cholangiocarcinoma: a case-control study in China. World J Gastroenterol 2011;17:249-253.

20. Fwu CW, Chien YC, You SL, Nelson KE, Kirk GD, Kuo HS, Feinleib M, et al. Hepatitis B virus infection and risk of intrahepatic cholangiocarcinoma and non-Hodgkin lymphoma: a cohort study of parous women in Taiwan. Hepatology 2011;53:1217-1225.

21. Welzel TM, Graubard BI, Zeuzem S, El-Serag HB, Davila JA, McGlynn KA. Metabolic syndrome increases the risk of primary liver cancer in the United States: a study in the SEER-Medicare database. Hepatology 2011;54:463-471.

22. Wu Y, Wang T, Ye S, Zhao R, Bai X, Wu Y, Abe K, et al. Detection of hepatitis B virus DNA in paraffin-embedded intrahepatic and extrahepatic cholangiocarcinoma tissue in the northern Chinese population. Hum Pathol 2012;43:56-61.

23. Liu ZY, Zhou YM, Shi LH, Yin ZF. Risk factors of intrahepatic cholangiocarcinoma in patients with hepatolithiasis: a case-control study. Hepatobiliary Pancreat Dis Int 2011;10:626-631.

24. Gong Y, Yang YS, Zhang XM, Su M, Wang J, Han JD, Guo MZ. ABO blood type, diabetes and risk of gastrointestinal cancer in northern China. World J Gastroenterol 2012;18:563-569.

25. Qu Z, Cui N, Qin M, Wu X. Epidemiological survey of biomarkers of hepatitis virus in patients with extrahepatic cholangiocarcinomas. Asia Pac J Clin Oncol 2012;8:83-87.

26. Wu Q, He XD, Yu L, Liu W, Tao LY. The metabolic syndrome and risk factors for biliary tract cancer: a case-control study in China. Asian Pac J Cancer Prev 2012;13:1963-1969.

27. Chaiteerakij R, Yang JD, Harmsen WS, Slettedahl SW, Mettler TA, Fredericksen ZS, Kim WR, et al. Risk factors for intrahepatic cholangiocarcinoma: association between metformin use and reduced cancer risk. Hepatology 2013;57:648-655.

28. Fedirko V, Lukanova A, Bamia C, Trichopolou A, Trepo E, Nothlings U, Schlesinger S, et al. Glycemic index, glycemic load, dietary carbohydrate, and dietary fiber intake and risk of liver and biliary tract cancers in Western Europeans. Ann Oncol 2013;24:543-553.

29. Zhou Y, Zhou Q, Lin Q, Chen R, Gong Y, Liu Y, Yu M, et al. Evaluation of risk factors for extrahepatic cholangiocarcinoma: ABO blood group, hepatitis B virus and their synergism. Int J Cancer 2013;133:1867-1875.

30. Chang JS, Tsai CR, Chen LT. Medical risk factors associated with cholangiocarcinoma in Taiwan: a population-based case-control study. PLoS One 2013;8:e69981.

31. Lee BS, Cha BH, Park EC, Roh J. Risk factors for perihilar cholangiocarcinoma: a hospital-based case-control study. Liver Int 2015;35:1048-1053.

32. Lee BS, Park EC, Park SW, Nam CM, Roh J. Hepatitis B virus infection, diabetes mellitus, and their synergism for cholangiocarcinoma development: a case-control study in Korea. World J Gastroenterol 2015;21:502-510.

33. Peng YC, Lin CL, Hsu WY, Chang CS, Yeh HZ, Tung CF, Wu YL, et al. Statins are associated with a reduced risk of cholangiocarcinoma: a population-based case-control study. Br J Clin Pharmacol 2015;80:755-761.

34. Choi J, Ghoz HM, Peeraphatdit T, Baichoo E, Addissie BD, Harmsen WS, Therneau TM, et al. Aspirin use and the risk of cholangiocarcinoma. Hepatology 2016;64:785-796.

35. Meng ZW, Han SH, Zhu JH, Zhou LY, Chen YL. Risk Factors for Cholangiocarcinoma After Initial Hepatectomy for Intrahepatic Stones. World J Surg 2017;41:835-843.

36. Kamiza AB, Su FH, Wang WC, Sung FC, Chang SN, Yeh CC. Chronic hepatitis infection is associated with extrahepatic cancer development: a nationwide population-based study in Taiwan. BMC Cancer 2016;16:861.

37. Huang YJ, Wu AT, Chiou HY, Chuang MT, Meng TC, Chien LN, Yen Y. Interactive role of diabetes mellitus and female sex in the risk of cholangiocarcinoma: A population-based nested case-control study. Oncotarget 2017;8:6642-6651.

38. Mahale P, Torres HA, Kramer JR, Hwang LY, Li R, Brown EL, Engels EA. Hepatitis C virus infection and the risk of cancer among elderly US adults: A registry-based case-control study. Cancer 2017;123:1202-1211.

39. Wei XL, Luo HY, Li CF, Jin Y, Zeng ZL, Ju HQ, Wu QN, et al. Hepatitis B virus infection is associated with younger median age at diagnosis and death in cancers. Int J Cancer 2017;141:152-159.

40. Petrick JL, Yang B, Altekruse SF, Van Dyke AL, Koshiol J, Graubard BI, McGlynn KA. Risk factors for intrahepatic and extrahepatic cholangiocarcinoma in the United States: A population-based study in SEER-Medicare. PLoS One 2017;12:e0186643.

41. An J, Kim JW, Shim JH, Han S, Yu CS, Choe J, Lee D, et al. Chronic hepatitis B infection and non-hepatocellular cancers: A hospital registry-based, case-control study. PLoS One 2018;13:e0193232.

42. Mahale P, Engels EA, Koshiol J. Hepatitis B virus infection and the risk of cancer in the elderly US population. Int J Cancer 2019;144:431-439.

43. Peng YC, Lin CL, Hsu WY, Chow WK, Lee SW, Yeh HZ, Chen CC, et al. Association Between Cholangiocarcinoma and Proton Pump Inhibitors Use: A Nested Case-Control Study. Front Pharmacol 2018;9:718.

44. Xiong J, Lu X, Xu W, Bai Y, Huang H, Bian J, Zhang L, et al. Metabolic syndrome and the risk of cholangiocarcinoma: a hospital-based case-control study in China. Cancer Manag Res 2018;10:3849-3855.

45. Zhou Z, Nie SD, Jiang B, Wang J, Lv P. Risk factors for extrahepatic cholangiocarcinoma: a case-control study in China. Eur J Cancer Prev 2019;28:254-257.

46. Lavu S, Therneau TM, Harmsen WS, Mara KC, Wongjarupong N, Hassan M, Ali HA, et al. Effect of Statins on the Risk of Extrahepatic Cholangiocarcinoma. Hepatology 2020;72:1298-1309.

47. Xiong J, Wang Y, Chen G, Jin L. Proton pump inhibitors and the risk of gallbladder cancer: a hospital-based case-control study. Gut 2020;69:2265-2267.

48. Tian T, Song C, Jiang L, Dai J, Lin Y, Xu X, Yu C, et al. Hepatitis B virus infection and the risk of cancer among the Chinese population. Int J Cancer 2020;147:3075-3084.

49. Uenishi T, Hirohashi K, Kubo S, Yamamoto T, Yamazaki O, Kinoshita H. Clinicopathological factors predicting outcome after resection of mass-forming intrahepatic cholangiocarcinoma. Br J Surg 2001;88:969-974.

50. Asayama Y, Aishima S, Taguchi K, Sugimachi K, Matsuura S, Masuda K, Tsuneyoshi M. Coexpression of neural cell adhesion molecules and bcl-2 in intrahepatic cholangiocarcinoma originated from viral hepatitis: relationship to atypical reactive bile ductule. Pathol Int 2002;52:300-306.

51. Hai S, Kubo S, Yamamoto S, Uenishi T, Tanaka H, Shuto T, Takemura S, et al. Clinicopathologic characteristics of hepatitis C virus-associated intrahepatic cholangiocarcinoma. Dig Surg 2005;22:432-439.

52. Zhang L, Cai JQ, Zhao JJ, Bi XY, Tan XG, Yan T, Li C, et al. Impact of hepatitis B virus infection on outcome following resection for intrahepatic cholangiocarcinoma. J Surg Oncol 2010;101:233-238.

53. Zhou HB, Wang H, Li YQ, Li SX, Wang H, Zhou DX, Tu QQ, et al. Hepatitis B virus infection: a favorable prognostic factor for intrahepatic cholangiocarcinoma after resection. World J Gastroenterol 2011;17:1292-1303.

54. Jiang BG, Ge RL, Sun LL, Zong M, Wei GT, Zhang YJ. Clinical parameters predicting survival duration after hepatectomy for intrahepatic cholangiocarcinoma. Can J Gastroenterol 2011;25:603-608.

55. Wu ZF, Yang N, Li DY, Zhang HB, Yang GS. Characteristics of intrahepatic cholangiocarcinoma in patients with hepatitis B virus infection: clinicopathologic study of resected tumours. J Viral Hepat 2013;20:306-310.

56. Li T, Qin LX, Zhou J, Sun HC, Qiu SJ, Ye QH, Wang L, et al. Staging, prognostic factors and adjuvant therapy of intrahepatic cholangiocarcinoma after curative resection. Liver Int 2014;34:953-960.

57. Liu RQ, Shen SJ, Hu XF, Liu J, Chen LJ, Li XY. Prognosis of the intrahepatic cholangiocarcinoma after resection: hepatitis B virus infection and adjuvant chemotherapy are favorable prognosis factors. Cancer Cell Int 2013;13:99.

58. Luo X, Yuan L, Wang Y, Ge R, Sun Y, Wei G. Survival outcomes and prognostic factors of surgical therapy for all potentially resectable intrahepatic cholangiocarcinoma: a large single-center cohort study. J Gastrointest Surg 2014;18:562-572.

59. Uenishi T, Nagano H, Marubashi S, Hayashi M, Hirokawa F, Kaibori M, Matsui K, et al. The long-term outcomes after curative resection for mass-forming intrahepatic cholangiocarcinoma associated with hepatitis C viral infection: a multicenter analysis by Osaka Hepatic Surgery Study Group. J Surg Oncol 2014;110:176-181.

60. Zhang GW, Lin JH, Qian JP, Zhou J. Identification of risk and prognostic factors for patients with clonorchiasis-associated intrahepatic cholangiocarcinoma. Ann Surg Oncol 2014;21:3628-3637.

61. Ahn CS, Hwang S, Lee YJ, Kim KH, Moon DB, Ha TY, Song GW, et al. Prognostic impact of hepatitis B virus infection in patients with intrahepatic cholangiocarcinoma. ANZ J Surg 2018;88:212-217.

62. Pan QX, Su ZJ, Zhang JH, Wang CR, Ke SY. Glasgow Prognostic Score predicts prognosis of intrahepatic cholangiocarcinoma. Mol Clin Oncol 2017;6:566-574.

63. Jeong S, Cheng Q, Huang L, Wang J, Sha M, Tong Y, Xia L, et al. Risk stratification system to predict recurrence of intrahepatic cholangiocarcinoma after hepatic resection. BMC Cancer 2017;17:464.

64. Yuan L, Luo X, Lu X, Yi B, Chu K, Cai Q, Jiang X. Comparison of clinicopathological characteristics between cirrhotic and non-cirrhotic patients with intrahepatic cholangiocarcinoma: A large-scale retrospective study. Mol Clin Oncol 2017;7:615-622.

65. Jeong S, Gao L, Tong Y, Xia L, Xu N, Sha M, Zhang J, et al. Prognostic Impact of Cirrhosis in Patients with Intrahepatic Cholangiocarcinoma following Hepatic Resection. Can J Gastroenterol Hepatol 2017;2017:6543423.

66. Chae H, Cho H, Yoo C, Kim KP, Jeong JH, Chang HM, Kang J, et al. Prognostic implications of hepatitis B virus infection in intrahepatic cholangiocarcinoma treated with first-line gemcitabine plus cisplatin. Int J Biol Markers 2018;33:432-438.

67. Lu JC, Zeng HY, Sun QM, Meng QN, Huang XY, Zhang PF, Yang X, et al. Distinct PD-L1/PD1 Profiles and Clinical Implications in Intrahepatic Cholangiocarcinoma Patients with Different Risk Factors. Theranostics 2019;9:4678-4687.

68. Ge Y, Jeong S, Luo GJ, Ren YB, Zhang BH, Zhang YJ, Shen F, et al. Transarterial chemoembolization versus percutaneous microwave coagulation therapy for recurrent unresectable intrahepatic cholangiocarcinoma: Development of a prognostic nomogram. Hepatobiliary Pancreat Dis Int 2020;19:138-146.

69. Kaibori M, Yoshii K, Kashiwabara K, Kokudo T, Hasegawa K, Izumi N, Murakami T, et al. Impact of hepatitis C virus on survival in patients undergoing resection of intrahepatic cholangiocarcinoma: report of a Japanese nationwide survey. Hepatol Res 2021.
